# Macular Function in Early and Intermediate Age-related Macular Degeneration: Correlation with the Simplified Thea Risk Assessment Scale (STARS)

**DOI:** 10.1167/tvst.9.10.28

**Published:** 2020-09-28

**Authors:** Angelo Maria Minnella, Marco Piccardi, Giorgio Placidi, Alfredo García-Layana, Cecile Delcourt, Patrizia Valentini, Benedetto Falsini

**Affiliations:** 1 Ophthalmology, Università Cattolica del Sacro Cuore, Rome, Italy; 2 Fondazione Policlinico Universitario A. Gemelli- IRCCS, Rome, Italy; 3 Ophthalmology Department, Clínica Universidad de Navarra, University of Navarra, Pamplona, Spain; 4 University of Bordeaux, Inserm, Bordeaux Population Health Research Center, UMR 1219, Team LEHA, Bordeaux, France

**Keywords:** age-related macular degeneration, focal electroretinogram; risk factors; visual acuity

## Abstract

**Purpose:**

Early detection of retinal dysfunction in age-related macular degeneration (AMD) may be important for both prevention and treatment. The aim of this study was to evaluate in early and intermediate AMD the correlation of macular function, assessed by the focal electroretinogram (fERG), with the Simplified Thea Risk Assessment Scale (STARS), a simple 13-item self-administered questionnaire.

**Methods:**

We recorded a fERG (18°, 41 Hz) in 84 patients with AMD (40 male and 44 female, age 55–87 years, visual acuity 20/40–20/20), who had undergone a 5-year clinical ophthalmic and general follow-up. Sixty-six patients had early and 17 patients intermediate AMD. Fifty healthy subjects, in a comparable age range, served as controls. The fERG amplitude (in microVolts) was the main outcome variable. STARS was calculated for each patient.

**Results:**

Compared with controls, fERG amplitudes were significantly reduced, on average, in both early and intermediate patients with AMD (*P* < 0.01). In both groups, fERG amplitudes tended to decrease with age and to increase with visual acuity and were negatively correlated with STARS (early r = –0.6, *P* < 0.01; intermediate, r = –0.50, *P* < 0.05). fERG losses were greatest in patients with a STARS score of greater than 20.

**Conclusions:**

In early and intermediate AMD, STARS robustly predicted central retinal function, as assessed by fERG, supporting the combined use of both parameters to estimate the clinical risk of visual function loss.

**Translational Relevance:**

The STARS may predict macular function in AMD and could be used in the daily clinical practice to estimate the risk of visual function loss in early disease stages.

## Introduction

Age-related macular degeneration (AMD), particularly neovascular AMD, is one of the most common causes of blindness worldwide in individuals older than 60 years of age.^1^ Recently, the prevalence of early AMD in Europe was found to increase from 3.5% in subjects aged 55 to 59 years to 17.6% in those aged 85 years and older; for late AMD, the prevalence increase was from 0.1% to 9.8%, respectively.^2^ All these changes might result by 2040 in an almost doubling of affected persons, with a number of individuals with early AMD between 14.9 and 21.5 million, and with late AMD between 3.9 and 4.8 million.

Several risk factors such as age and smoking have been associated with AMD.^3^^–^^9^ A meta-analysis showed strong and consistent associations with advancing age, current cigarette smoking, previous cataract surgery, and family history of AMD.^10^ Moreover, higher body mass index, a history of cardiovascular disease, hypertension, and increased fibrinogen levels were moderately and consistently associated with AMD. Conversely, relationships between changes in cholesterol levels and AMD have been less consistent, although pooled data from patients of European ancestry showed that total serum cholesterol was inversely associated with the prevalence of early AMD.^1^^,^^11^ The Simplified Thea Risk Assessment Scale (STARS) is a simple, easy-to-use, self-administered 13-item questionnaire to assess personalized risk for AMD in routine clinical practice.^12^ The questionnaire was established through a scoring system derived from a sample of more than 12,000 subjects and validated in more than 6.000, subjects.

A focal electroretinogram (fERG) is a tool for diagnosis, analysis of pathogenesis, prediction of prognosis, and evaluation of central retinal function in several macular diseases. A fERG can be recorded from the macular region in response to a flickering stimulus presented on a light adapting background to minimize the stray-light modulation on peripheral retina. This relatively simple methodology was introduced for the first time by Seiple et al.,^13^ and has been widely used to assess macular function in several retinal degenerative diseases, including AMD. In a cross-sectional study, significant amplitude losses and phase delays of the 41 Hz fERG responses in early and intermediate AMD were observed.^14^ Furthermore, the fERG to 41 Hz stimuli provided a sensitive measure of retinal function changes induced by antioxidant treatment in early AMD.^15^

The detection of early visual loss in early AMD is important for both prevention and treatment. The aim of this study was to evaluate in early and intermediate AMD the correlation of macular function, assessed by fERG, and the AMD risk, assessed by using the STARS questionnaire.

## Methods

### Study Design and Participants

This single-center study was conducted at Fondazione Policlinico Universitario A. Gemelli – IRCCS, according to the principles of the Declaration of Helsinki, and complied with Good Clinical Practices and received before study initiation approval from relevant national and local ethic committees. Because this was a noncomparative trial, no trial registration was made. A total of 83 patients with AMD and 50 healthy subjects serving as the control group were included between 2012 and 2013. All patients had undergone a previous 5-year clinical ophthalmic follow up. Moreover, subjects with or without non–insulin-dependent diabetes (NIDD) were suitable for inclusion. Subjects with neovascularization, geographic atrophy, glaucoma, uveitis, or media opacity were excluded from this study. According to the Ferris et al. classification,^16^ early AMD was defined by the presence of medium drusen (>63 µm and <125 µm) and no AMD pigmentary abnormalities, whereas intermediate AMD was defined by the presence of large drusen (>125 µm) and/or any AMD pigmentary abnormalities.

### Procedures

The examination protocol included visual acuity (VA; according to the standard Snellen chart), fundus examination, fundus imaging, spectral domain optical coherence tomography (Cirrus, Carl Zeiss Meditec, Inc., Dublin, CA) and fERG.

The fERG was recorded from the central 18° region using a uniform red field stimulus superimposed on an equiluminant steady adapting background used to minimize stray light modulation. The stimulus was generated by a circular array of eight red LEDs (k maximum 660 nm, mean luminance of 93 cd/m^2^) presented on the rear of a Ganzfeld bowl (white-adapting background, luminance of 40 cd/m^2^). A diffusing filter in front of the LED array made it appear as a circle of uniform red light. The fERGs were recorded in response to the sinusoidal 95% luminance modulation of a red uniform field (subtending 18° of visual angle) and centered on the fovea. Flickering frequency was 41 Hz. The same apparatus was used throughout the years, with periodic controls of LED and background intensity. Under the constant monitoring of an external observer, patients fixed a central fixation mark monocularly at 0.25°. Pupils were dilated to a diameter of 8 mm using 1% tropicamide and 2.5% phenylephrine hydrochloride, and all subjects underwent a preadaptation period of 20 minutes to the stimulus mean illuminance. The fERGs were recorded by an Ag–AgCl electrode taped on the skin over the lower eyelid. A similar electrode, placed over the eyelid of the contralateral patched eye, was used as reference (interocular recording). The fERG signals were amplified (100,000-fold), bandpass filtered between 1 and 100 Hz (6 decibels/octave), and averaged (12-bit resolution, 2-kHz sampling rate, 200–600 repetitions in 2–6 blocks). Off-line discrete Fourier analysis quantified the peak-to-peak amplitude and phase lag of the response fundamental harmonic (first harmonic) at 41 Hz. The lower limit of the fERG amplitude normal range was 1.1 µV.

Patients completed the STARS questionnaire and a score was derived.^12^ As reported by Delcourt et al.,^12^ the questionnaire was designed to be simple, fast, and easy. The risk factors included age, sex, body mass index, ethnicity, family history of AMD, smoking, personal medical history (systemic hypertension, clinical history of myocardial infarction, hypercholesterolemia), and eye-related parameters (iris color, cataract, refraction). Although it was not planned to deliver the STARS questionnaire to normal control subjects, a subgroup (*n* = 10) agreed to complete the questionnaire and a score was calculated also for them. The data were collected on a single form with demography, medical history, and lifestyle questions filled in by the patient.

### Statistical Methods

Measurements resulting from the right eyes of each patient were included in the main statistical analysis. The fERG amplitudes and phase were compared across controls, patients with early AMD, and patients with intermediate AMD by analysis of variance adjusted *t*-tests for post hoc multiple comparisons. A multiple stepwise regression was performed to evaluate the association of the fERG parameter amplitude and phase of patients with the STARS score. The model included fERG amplitude and phase as dependent variables and STARS score and age as independent variables. Pearson's correlations were used to evaluate, separately, the association of fERG amplitude with age and VA. For all analyses, a *P* value of less than 0.05 was considered as statistically significant.

## Results

Overall, 66 subjects with early AMD, 17 with intermediate AMD, and 50 healthy subjects were included. All subjects were aged between 55 and 87 years. Patient demographic data and STARS questionnaire score results are summarized in Table 1. The mean age tended to increase in patients with intermediate AMD compared with patients with early AMD. Acuity was decreased in both early and intermediate patients with AMD compared with controls. There was also a significant difference between the two groups of patients. A substantial proportion of patients with early AMD and patients with intermediate AMD had systemic hypertension, although few had NIDD and a smoking history. As expected, the STARS score tended to increase in patients with intermediate AMD compared with patients with early AMD. The significance of these differences is reported in detail in Table 1.

**Table 1.** Summary of Demographic and Clinical Data of Control Patients and Patients with AMD

Diagnosis	*N* (Male/Female)	Age (Mean, SD)	Acuity (LogMAR) (mean, SD)	Systemic Hypertension (yes/no)	Diabetes (yes/no)	Smoking (yes/no)	STARS (Mean, SD)
Normal	50 (25/25)	69 (8)	0.0	na	na	na	na
Early AMD	66	67.8 (8)	0.097 (0.01)^*^	40/26	16/50	20/46	12(4)
Intermediate AMD	17	72.8 (8)	0.4 (0.1)^**^	8/9	9/8	8/9	16.6 (6)^#^

LogMAR, logarithm of the minimum angle of resolution; na, not applicable; SD, standard deviation.

* Significantly reduced compared with controls (*P* = 0.035).

** Significantly reduced compared with controls (*P* = 0.015) and to early AMD (*P* = 0.035).

# Significantly reduced compared with early AMD (*P* = 0.04).

**Figure 1.**
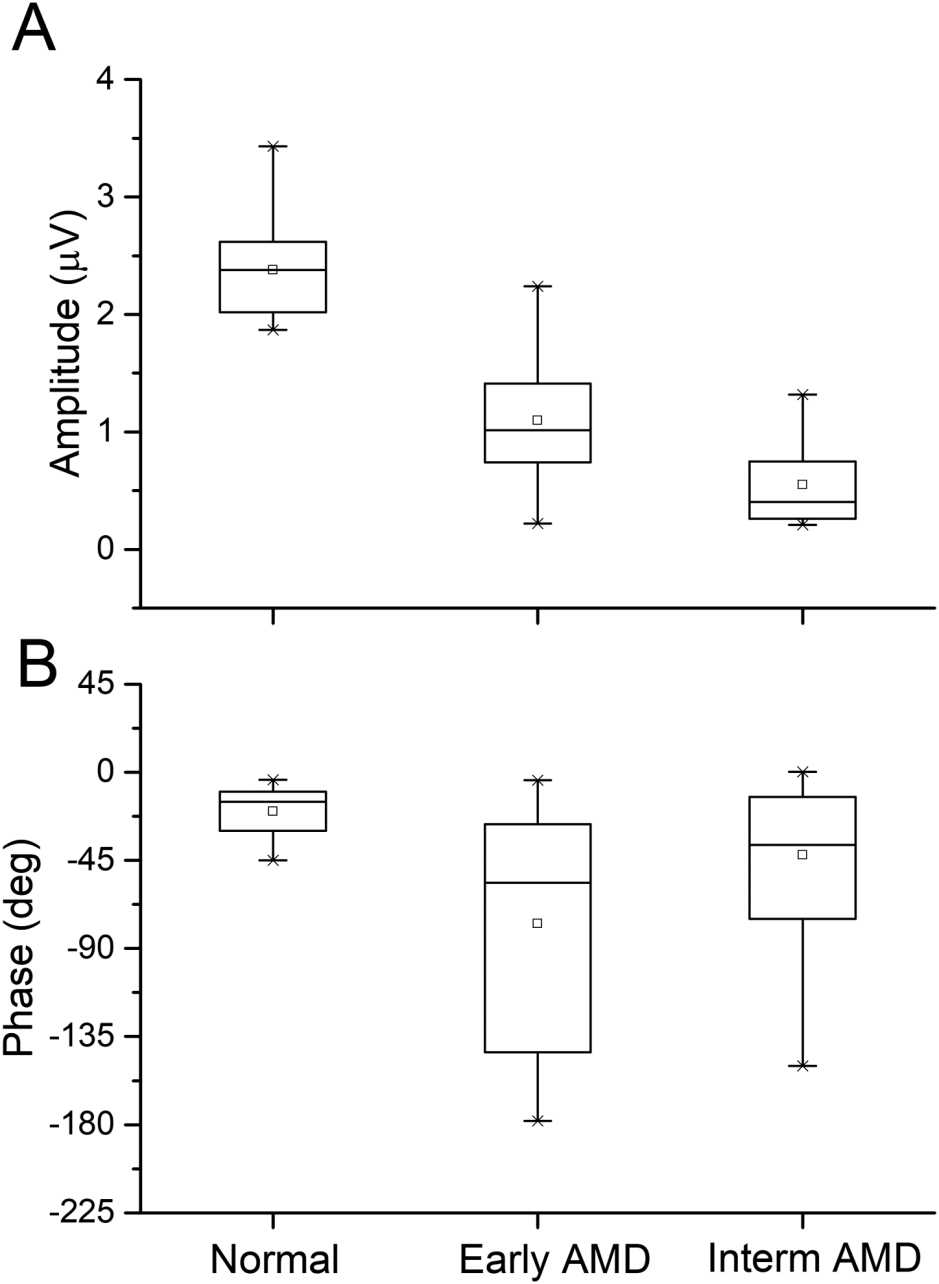
Box plot analyses of fERG amplitude (A) and phase (B) in controls (Normal), patients with early AMD and patients with intermediate AMD. Analysis of variance: fERG amplitude was decreased (f-ratio = 75; *df* 3,97; *P* = 0.0009) and phase was delayed (f-ratio = 9; *df* 3,97; *P* = 0.008) across. Post hoc adjusted *t*-tests revealed significant differences between both groups of patients and controls (*P* = 0.0095) and for fERG amplitudes between early and intermediate patients with AMD (*P* = 0.048).

The fERG amplitudes and phases were recorded in control subjects, patients with early AMD, and patients with intermediate AMD (Fig. 1). Analysis of variance showed that fERG amplitude was decreased (f-ratio = 75; *df* 3,97; *P* = 0.0009) and phase was delayed (f-ratio = 9; *df* 3,97; *P* = 0.008) across groups. Post hoc adjusted *t*-tests revealed significant differences between both groups of patients and controls (*P* = 0.0095) and for fERG amplitudes between patients with early AMD and patients with intermediate AMD (*P* = 0.048).

**Figure 2.**
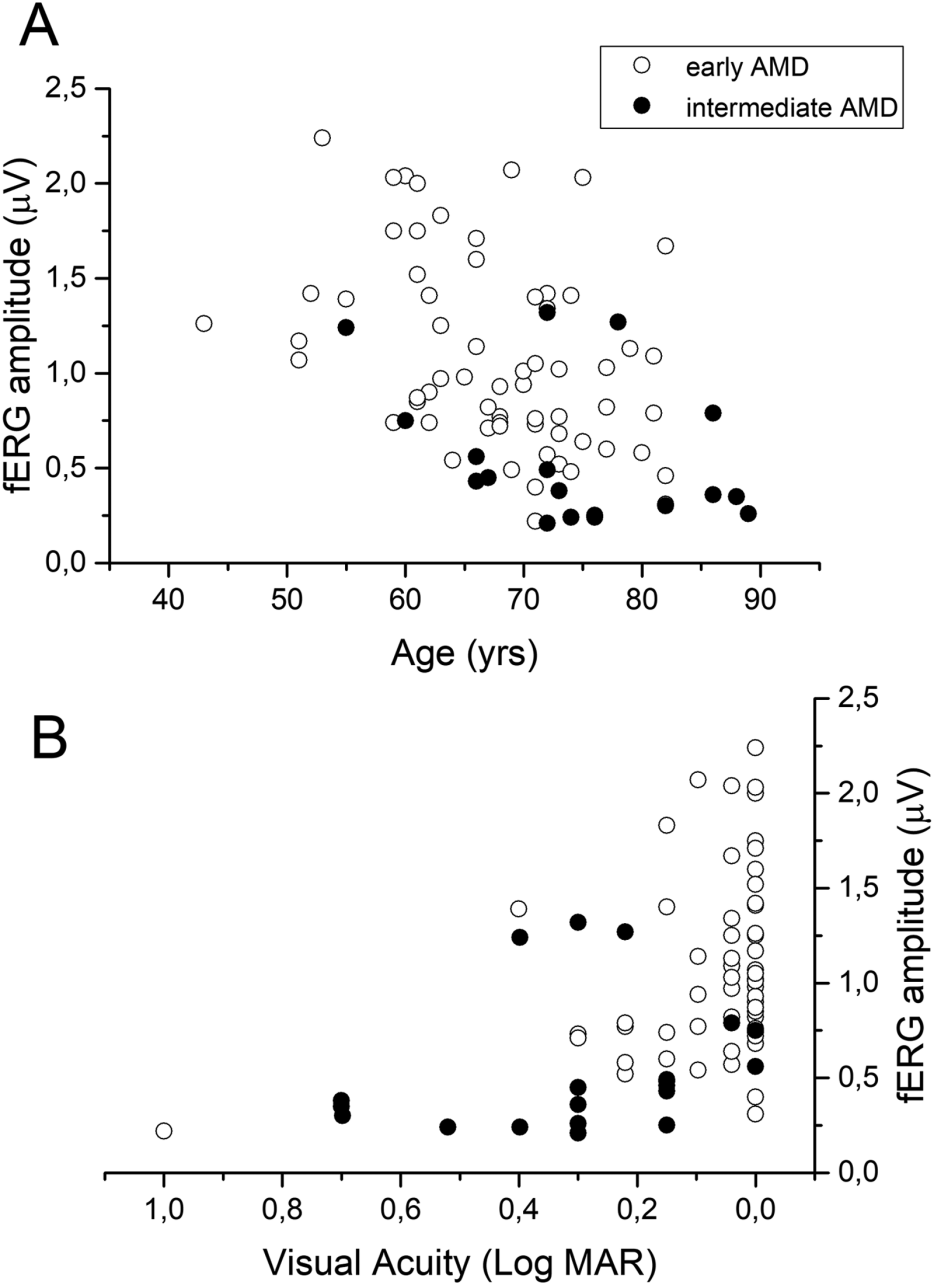
fERG amplitude plotted as a function of age (A) and VA (logarithm of the minimum angle of resolution) (B), in subjects with early and intermediate AMD. fERG amplitude versus age (early AMD: r = –0.55, *P* = 0.007; intermediate AMD: r = –0.48, *P* = 0.045); and fERG amplitude versus VA (logarithm of the minimum angle of resolution) (r = 0.48, *P* = 0.009).

Scatterplots of fERG amplitudes plotted as a function of age (A) and VA (B) were shown in Figure 2. The fERG amplitudes tended to decrease significantly with age and to increase with VA (fERG amplitude versus age: early AMD, r = –0.55, *P* = 0.007; intermediate AMD, r = –0.48, *P* = 0.045); fERG amplitude versus VA (logarithm of the minimum angle of resolution) (r = 0.48, *P* = 0.009). In control subjects, fERG amplitude, but not phase, also tended to decrease with age (r = 0.46, *P* = 0.001). The rate of amplitude decrease with age in the range of 55 to 80 years, expressed by the slope of the linear regression, was similar between control and patients (–0.02 µV/year).

The fERG phase did not show a significant correlation with age and VA. No significant correlation with the presence of systemic hypertension, diabetes or smoking was observed. Patients with NIDD tended to have lower amplitude (25%) and a delayed phase (–30°) compared with nondiabetic patients. However, this difference did not attain statistical significance (*P* = 0.07).

**Figure 3.**
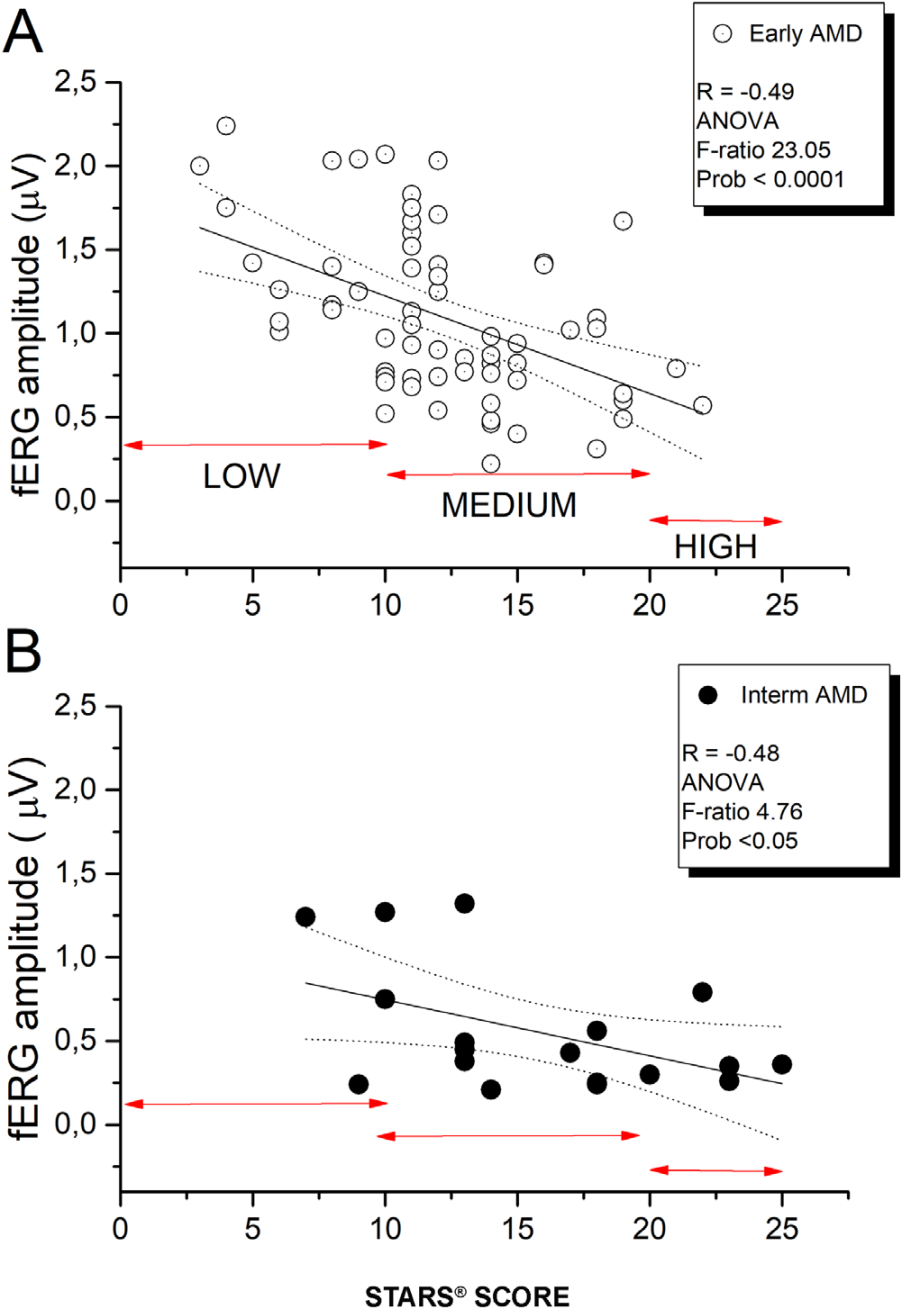
fERG amplitude plotted as a function of STARS score for patients with early AMD (A) and patients with intermediate AMD (B). In both groups, linear regression analysis reveals a significant association between the two parameters. fERG amplitude versus the STARS score: early AMD (*P* = 0.00008), intermediate AMD (*P* = 0.043). *Arrows* in A indicate low-, medium-, and high-risk scores. *Arrows* in B indicate the same score groups as in A. ANOVA, analysis of variance.

Figure 3 shows the fERG amplitudes of patients with early AMD (Fig. 3A) and patients with intermediate AMD (Fig. 3B) plotted as a function of the STARS score. A significant negative correlation between the fERG amplitude and the STARS score for either patients with early AMD (*P* = 0.00008) or patients with intermediate AMD (*P* = 0.043) was observed. Stepwise multiple regression analysis showed a significant association between fERG amplitude and the STARS score (*P* < 0.0001). The fERG phase did not show a significant correlation with the STARS. In Figures 3A and 3B, arrows designate the range for a low, medium, and high risk of the STARS.

A multiple regression analysis was also carried out on individual items of the questionnaire as independent variable and fERG amplitude as dependent variable. It was found that, in early AMD, after correction for age, the overall effect was statistically highly significant (*P* = 0.0006). In addition, fruit consumption turned out to be protective on retinal function (*P* = 0.02). In intermediate AMD, none of the individual items reached the statistical significance, although the total score was significantly correlated with fERG amplitude (*P* = 0.043). Table 2 reports the details of multiple regression for each item in the two patient groups. Ethnicity was not included because the patients were all Caucasian.

**Table 2.** Results of Multiple Regression Analysis of Multiple Items of Questionnaire and fERG Amplitude as Dependent Variable in Patients with AMD

Early					
		Value	SE	t-Value	Prob>|t|
Intercept		1.17	0.46	2.40	0.02^*^
	Sex	–0.04	0.11	–0.37	0.71
	Age	–0.14	0.04	–3.71	0.00^*^
	BMI	–0.06	0.10	–0.61	0.53
	Com.	–0.00	0.02	–0.26	0.79
	Family	–0.03	0.02	–1.59	0.11
	Smok	–0.09	0.05	–1.79	0.08
	Cat	–0.05	0.02	–1.72	0.09
	Refr	0.02	0.03	0.66	0.50
	Vegetable	–0.01	0.27	–0.06	0.95
	Fruit	–0.55	0.23	–2.38	0.02^*^
	Fish	–0.02	0.11	–0.22	0.82
Overall effect	*P* = 0.0006				
Intermediate					
Intercept		–0.00	0	–0.15	0.88
	Sex	0.34	0.34	1.00	0.35
	Age	–0.08	0.06	–1.53	0.17
	BMI	–0.49	0.37	–1.31	0.23
	Com.	0.04	0.07	0.60	0.57
	Family	–0.08	0.07	–1.05	0.33
	Smok	0.06	0.23	0.27	0.79
	Cat	0.08	0.10	0.72	0.49
	Refr	–0.11	0.09	–1.20	0.27
	Vegetable	0.00	0.00	–0.15	0.88
	Fruit	0.03	0.54	0.05	0.96
	Fish	0.61	0.37	1.66	0.14
Overall effect	*P* = 0.043				

BMI, body mass index; Cat, presence or absence of cataract; com., comorbidities; Refr, presence of refractive error; SE, standard error of the mean; Smok, smoking history.

* Statistically significant.

**Figure 4.**
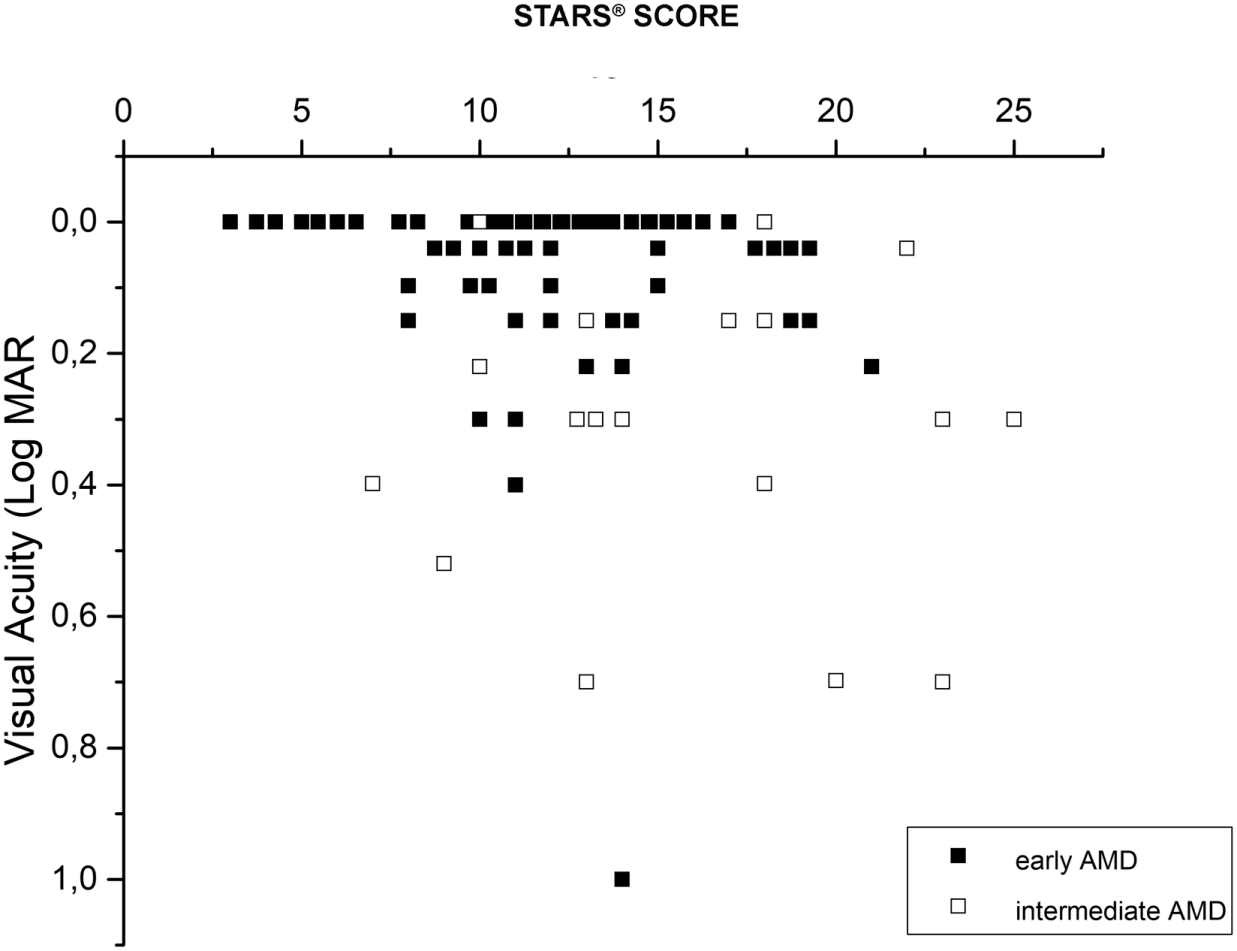
Scatterplot of VA as a function of the STARS score in early (*filled squares*) and intermediate (*open squares*) patients with AMD (VA versus STARS score [pooled data] r = 0.27; *P* = 0.04).

Figure 4 shows a scatter of VA as a function of the STARS for early and intermediate AMD. In both groups of patients, VA tended to decrease with increasing STARS score values. When performing a correlation analysis separately for the two groups, no significant correlation between the VA and STARS score was found. When pooling the data, the trend reached statistical significance (r = 0,27, *P* = 0.04). However, the strength of the correlation was weaker than that observed for the fERG amplitude (Fig. 3A). In addition, overlap between visual acuities of low- and high-risk eyes was much larger, suggesting lower diagnostic discrimination.

## Discussion

The identification of early biomarkers of retinal and visual dysfunction in patients with AMD has become of increasing importance regarding novel, personalized, and early disease treatments.^17^^,^^18^ Our study evaluated the relationship of the central retinal function in patients with early and intermediate AMD, assessed by the fERG, with systemic risk factors, as summarized by the STARS questionnaire.

The multiple regression analysis showed a significant negative correlation between the amplitude of the fERG and the STARS score. Age was not included in the model because its effect did not increase the strength of the association significantly, although fERG amplitude decreased significantly as a function of age.

In Figure 3A, there was only a minimal overlap (1 of 13 patients with a low risk score) in fERG amplitude between low- and high-risk eyes, suggesting a relatively high discrimination between these two groups of AMD eyes, as classified on the basis of STARS. Although the number of patients with early AMD with high-risk scores was too limited to draw robust conclusions, the data in Figure 3B from patients with intermediate AMD confirm that high risk scores are always associated with low amplitudes of the fERG. The association was even more robust than that between VA and the same score. Regarding the correlation of STARS score with fERG amplitude, the best association was found for patients with early AMD. In intermediate patients with AMD, there still was a statistical correlation between STARS and fERG amplitude. However, the correlation was less strong. We believe this was related to two reasons: (1) The smaller sample of patients with intermediate AMD versus patients with early AMD, and (2) a floor effect related to the low amplitude of the fERG and the higher STARS score of individual patients with intermediate AMD versus patients with early AMD. The main focus of the study was the correlation between retinal function and STARS score in patients with AMD. Therefore, the assessment of the STARS score in our group of normal subjects was beyond the scope of our study. However, we were able to measure STARS score in a subgroup of 10 normal subjects from our control group, and we found a value of 6 with a standard deviation of 2.

The fERG technique used in our study has been successfully used for decades by other groups in several clinical studies to test both physiologic and pathologic conditions.^13^^,^^19^ In addition, fERG was useful as outcome variable in clinical trials which aimed to test potential neuroprotective agents that delay and/or rescue photoreceptor damage in the early stages of AMD.^15^^,^^20^

As in previous studies, the fERG technique was a valid clinical method to estimate retinal flicker responses in normal retinal physiology as well as in patients with a degenerative disease of the outer retina. We considered that the use of such an approach may be useful to further advance research in AMD.^14^^,^^21^ In our study, fERG tested the function of the central 18° of the retina, including both macular and perimacular areas, where the disease usually manifests in early stages, with drusen and retinal pigment epithelium abnormalities. Interestingly, flicker sensitivity has been found to be predictive, more than VA, of late-onset AMD.^22^ Based on these data, we may suggest that flicker sensitivity, in our setting measured by fERG, can be more predictive of the retinal functional and anatomic status in individual patients with AMD. Clearly, further longitudinal studies are needed to confirm this hypothesis.

After correction for age and AMD stage, no significant effect on fERG results of our patients was observed for smoking or systemic hypertension. However, a subgroup of only 16 patients had NIDD (mean duration, 4 years; range, 1–10 years). Diabetes is a potentially important factor in determining visual loss in AMD. An indirect link between diabetes and AMD has been recently reported.^23^ In this population, the fERG amplitude tended to be reduced compared with the nondiabetic patient population. However, diabetes was not included as a factor in the statistical analysis, because the number of diabetic patients was too small. Removing the subgroup of diabetic patients from the statistical analysis did not change the significance of results. Nevertheless, future studies will have to focus on this parameter as a potential factor influencing macular cone function in AMD.

The clinical relevance of our study lies in the observed relationship between fERG amplitude results and the risk score of patients. STARS robustly predicted fERG responses of individual patients, that is, a patient with a high risk score was also more likely to show a decreased fERG amplitude, whereas a patient with a low score had most likely a normal fERG. Conversely, the same prediction was not as clear for VA, because a greater overlap between low- and high-risk eyes was observed. Moreover, in patients with a medium risk score there was a subgroup with an abnormally reduced fERG. These patients may belong to the high-risk group or their condition may evolve more rapidly toward a high risk score value.

Combining functional results and risk assessment is an approach that has been recently adopted by Flynn et al.^24^ These authors, recording the psychophysical recovery after bleaching, reported that the time to rod-mediated recovery in AMD eyes was associated with the disease severity stage, and may predict subsequent visual loss during the course of the disease. A similar predictive relationship was observed if the noninvasive fERG protocol used in this study is combined with the STARS score. Results from our study should prompt additional longitudinal studies to evaluate to potential predictive value of the combined functional and risk analysis in individual patients with AMD.

The main limitation of our study is its cross-sectional design. Therefore, the predictive value of our approach requires validation through a longitudinal study. Despite this limitation, the observed correlation between the electrophysiologic central retinal function and the risk of AMD is a novel finding requiring further investigations.

In conclusion, in early and intermediate AMD, central fERG amplitude robustly correlated with the disease risk score, supporting the combined use of both parameters to quantitatively estimate the risk of macular function loss. In particular, it is noteworthy that the value of STARS may predict macular function quite accurately in early stages of AMD. If the predictive value is confirmed in longitudinal studies, STARS may become a novel, easy, and reliable AMD biomarker.
